# Effect of propolis on membrane bound enzymes linked with breast cancer

**DOI:** 10.6026/973206300181192

**Published:** 2022-12-31

**Authors:** Sadhasivan Meghalatha Thazhathuputhenpurayil, Muninathan Natarajan

**Affiliations:** 1 Central Research Laboratory, Meenakshi Medical College Hospital and Research Institute, Meenakshi academy of higher education and research (MAHER), Kanchipuram Tamil Nadu, India-631552

**Keywords:** Cancer, ATPase enzymes, Na+/ K+-ATPase, Withaferin-A and Benz (a) Pyrene

## Abstract

According to estimates from the World Health Organization [WHO], in 2020, 685000 people worldwide died and 2.3 million women were diagnosed with breast cancer. Breast cancer will be the most common cancer in the world by the end of 2020, when 7.8 million women will still be living who had received a diagnosis within the previous five years. The current study was formulated to note the effects of ethanolic extract of Propolis and Withaferin-A on Breast tumor initiation by benzo(a)pyrene [B(a)p], and its effects on ATPase enzyme levels. Na+/ K+-ATPase, Mg2+-ATPase, and calcium ATPase enzyme activities were decreased in the erythrocyte membrane and tissues of breast cancer-bearing animals compared with control groups. The levels of enzyme activities were near normal in Propolis treated animals. It wasapparent that the beneficial effect of Propolis was primarily exerted during the initiation and post-initiation stage of B(a)p-induced breast carcinogenesis. Overall, the data indicated that Propolis has modulating effects when administered intra-peritoneally on breast cancer-bearing animals.

## Background:

Breast cancer is the most common cancer in women worldwide, affecting 10-20% of women. Incidence appears to be increasing globally, but mortality is declining [1]. In the world, breast cancer affects more women than any other type of cancer. According to a recent GLOBOCAN 2020, there will be 2.3 million new cancer cases worldwide in 2020, accounting for 11.7% of all new cancer cases [2]. Based on incidence data from 1998-2002, age is the main factor in breast cancer incidence. The most affected women in Japan and China are between the ages of 45 and 50, but in Indian women, as in Western women, the proportion is low in the same age group and increases with age [3]. The p-type ATPase family contains enzymes that move ions across the cytoplasmic membrane using the energy generated by ATP hydrolysis. This family includes Na/K-ATPase [NKA], Ca2+-ATPase, and H+/K+-ATPase [4]. NKA is essential for cell adhesion and has aberrant expression and activity that may contribute to the onset and spread of certain malignancies. According to several articles, malignant tissues express Na+/K+-ATPase subunits differently than their corresponding normal tissues, providing a potent diagnostic tool [5]. Withania somnifera, a herb with bioactive compounds like withaferin-A (WA), [6] has applications like anticoagulant, antipyretic, antioxidant [7, 8,9], antiproliferative, apoptosis, mitochondrial membrane depolarization, caspase activation, migration inhibition and G2/M cell cycle arrest in various cancerous cells [9]. The Effect Of Propolis On Membrane Bound Enzymes And Glycoprotein Levels In Benzo(a)Pyrene Induced Breast Carcinogenesis was investigated in Wistar rats.

## Materials and Methods:

The study site for the Wistar rat studies was the Central Research Laboratory of Meenakshi Medical College and Research Institute in Enathur, Kanchipuram. The research was conducted between October 2019 and June 2021. Institutional Animal Ethical Clearance (IAEC No. 003/2019) was attained on 03/07/2019. We purchased female Wistar rats weighing 150-200 g from the National Institute of Nutrition in Hyderabad, India. They were kept in a controlled environment with temperature and humidity on alternating 12-hour light/dark cycles. All of the rats were given access to unrestricted amounts of water as well as regular pellet food (Gold Mohr rat feed, Ms. Hindustan Lever Ltd., Mumbai). Benz (a) Pyrene, Withaferin-A, and Propolis were procured from Aldrich Sigma Chemical, Mumbai, and the remaining chemicals were from SRL Chemicals in Chennai. Membrane-bound enzymes like Na+/ K+-ATPase were estimated by Bonting (1970) [10], estimation of Mg2+-ATPase was performed by Ohinishi et al. (1982)[11], and calcium ATPase enzyme Hjerten and Pan (1983) [12].

## Experimental Procedure:

From the total number of animals, five groups of six each were formed (see PDF for details).

Group I: Non-diseased animal group fed with saline

Group II: Disease control group:- Animals treated with benzo(a) pyrene (20 mg diluted in 0.5 ml of sunflower oil and 0.5 ml of saline in two mammary pads using "air pouch method") were given the drug twice a week for three months.

Group III: Orally Withaferin A (30mg/kg b.wt, ) was administered to the Breast cancer-bearing animals weekly once for four weeks.

Group IV: Ethanolic extract of propolis was orally (50 mg/kg body weight) fed to the breast cancer-bearing animals for 30 days.

Group V: Both Withaferin A and ethanolic extract of propolis (as above) were administered to the breast cancer-bearing animals.

## Ethanolic extract of Propolis preparation:

With occasional shaking over four days at 37°C, Propolis was extracted in a hermetically sealed glass jar using 95 percent v/v ethanol. The ethanolic extract was first filtered through Whatman Filter Paper #4, and then heated to 60°C under reduced pressure before being evaporated in a rotary evaporator.

## Cancer induction:

## Procedure for the Air pouch technique:Cancer induction:

Air pouches were made in Wistar rats by employing the technique described by Arun et al. (1984). There are about 2 ml of air in the 5 ml syringe. It was sealed and autoclaved for 20 minutes at 15 psi. The sterile air from the syringe was carefully injected beneath the mammary fat pad to form a sterile air pouch. The air within the pouch was given a day to settle before administering the carcinogen.

## Application of a carcinogen:

After adding 0.5 ml of sterile saline and 0.5 ml of sunflower oil, a sterile vial containing 20 mg of B(a)P was weighed. The vial was stopped and vigorously vortexed in order to produce an emulsion that was evenly distributed. A single dose of B(a)P was injected into the air pouch. The tumor's growth was tracked daily up until the 90th day when it attained its largest size.

## Organ and blood collection:

All test rats were decapitated through the cervical method after the experiment. Before testing blood parameters, serum and plasma were separated from the blood during blood collection using ethylene diamine tetraacetic acid (EDTA). The liver and breast tissues were homogenized with a motor-driven, Teflon-coated homogenizer in a 0.1M Tris-HCl buffer at a pH of 7.4 to achieve a 10% homogenate.

## Results:

The administration of Withaferin A and Propolis together effectively suppressed breast cancer as evidenced by an increase in Na+/ K+-ATPase, Mg2+-ATPase, & calcium ATPase enzyme activities in erythrocyte membrane and tissues of breast cancer-bearing animals compared with control groups. This was in contrast to breast cancer-bearing animals treated with either Withaferin A or Propolis alone. Animals treated with propolis had enzyme activity levels that were close to normal.

Figure 1(see PDF) shows the effect of Withaferin-A & Propolis on the ATPase activities of erythrocyte membranes in control and experimental animals. The disease control animals were showing a decreased concentration of membrane-bound enzyme activities in comparison with the control group-I. Group III & IV shows an increase in ATPase enzyme levels when treated with WA & Propolis, respectively. A significant rise of all the membrane-bound ATPase enzymes [Na+/ K+-ATPase, Mg2+-ATPase, and calcium ATPase enzyme] levels(#p<0.001) was found in Group V compared to group-II, which was treated with both WA & Propolis.

Figure 2(see PDF) depicts how Withaferin-A and Propolis affect the liver ATPase enzyme activity in experimental and control mice. The concentration of hepatic enzyme activity was lower in disease-control animals when compared to the normal control group-I animals. When Group III & IV are treated with WA & Propolis, respectively, the levels of ATPase enzymes rise. When Group-V was compared to Group-II, which received both WA and Propolis treatment, all three membrane-bound ATPase enzymes-Na+/K+-ATPase, Mg2+-ATPase, and calcium ATPase enzyme-showed a substantial increase in levels (#p<0.001).

## Discussion:

The decrease in membrane-bound ATPase enzymes in malignant circumstances, such as Na+/K+-ATPase, Mg2+-ATPase, and calcium ATPase,may be due to an increase in cell proliferation. By using a supplement regimen or a metabolic procedure, the ATPase levels can be restored naturally. The combination therapy of Propolis and Withaferin A results in a considerable rise in ATPase levels despite the fact that Propolis and Withaferin A alone show great success in malignant conditions. Flavonoids, phenolic esters, fatty acids,alcohols, aliphatic and aromatic acids, terpenes, and beta-steroids are the principal chemical groups present in Propolis. Terpenoids also contribute to Propolis's distinctive smell, while flavonoids are primarily responsible for the pharmacological characteristics of Propolis [13and 14]. Propolone B and Propolonone A, two components of red Propolis, exhibited anti-proliferative properties against prostate cancer cells (MCF-7), breast cancer cells (U-251), and glioma cells (PC-3) [15]. A study conducted treating with Propolis has shown significant recovery of membrane-bound enzymes like ATPase [16]. A study by Rafael Zúñiga and colleagues (2020) shows a brand-new mechanism via which WFA lowers breast cancer cell viability and proliferation. Inhibition of TASK-3 channels is a component of this mechanism and also demonstrated how the TASK-3 channel is inhibited by the specific binding of WFA to the amino acid residues Phe125 and Leu197. The study also shows the direct effect between WFA and TASK-3 channels in the prevention of breast tumor cells' development and may have therapeutic uses in cancer [17]. As a result, membrane-bound enzymes play a crucial part in regulating and limiting the growth of cancer cells. Before using these natural sources as a treatment for various disorders, clinical trials and additional research are needed.

## Conclusion:

One of the most intriguing materials that honey bees generate is called Propolis. It has long been known to have antibacterial, antifungal, and immunomodulatory effects. Additionally, Propolis has an anti-cancer impact. The goal of the current study is to comprehend how Propolis and its constituents affect the prevention of cancer. To determine how WA and Propolis affected benzo (a)pyrene-induced breast cancer in Wister rats, enzyme-bound enzyme levels were assessed in all animal groups. More studies on the mechanism of Propolis on membrane-bound enzymes might be required. We draw the conclusion from the study that Withaferin A and Propolis may be used in combination therapy to lessen breast cancer symptoms and restore the mammary gland's structural integrity.
